# Animal contact-related nontyphoidal *Salmonella enterica* outbreaks in humans in the U.S. (2009–2022): serovar-specific temporal trends and associations with exposure sources and settings

**DOI:** 10.3389/fpubh.2026.1755882

**Published:** 2026-02-04

**Authors:** Hammad Ur Rehman Bajwa, Suman Bhowmick, Csaba Varga

**Affiliations:** 1 Department of Pathobiology, College of Veterinary Medicine, University of Illinois Urbana-Champaign, Urbana, IL, United States; 2 Carl R. Woese Institute for Genomic Biology, University of Illinois Urbana-Champaign, Urbana, IL, United States

**Keywords:** animal contact, co-occurrence, incidence, outbreak, Salmonella, serovars, temporal trend, United States

## Abstract

**Background:**

Nontyphoidal *Salmonella enterica* poses a public health risk. Investigating the associations between different serovars and their exposure sources and settings can guide prevention and control efforts.

**Methods:**

Surveillance data on animal contact-associated single-state outbreaks in the U.S., reported to the Centers for Disease Control and Prevention via the National Outbreak Reporting System (NORS) between 2009 and 2022, were analyzed. Descriptive statistics described the serovar-specific incidence rates and the distribution of exposure sources and settings. The co-occurrence patterns of serovars, exposure sources, and settings were evaluated using network analysis and multiple correspondence analysis. Serovar-specific temporal trends were assessed using locally estimated scatterplot smoothing (LOESS) and the Mann-Kendall test.

**Results:**

From 2009 to 2022, a total of 104 NTS outbreaks were reported. *Salmonella Typhimurium* was the most dominant serovar (*n* = 33; 31.73%), followed by *S*. I 4,[5],12:i:- (*n* = 15; 14.42%), and *S. enteritidis* (*n* = 10; 9.62%). Serovar Enteritidis was linked to poultry, while Typhimurium and I 4,[5],12:i:- were associated with mammals, reptiles, and agricultural settings. Among 32 serovars, only Montevideo showed a significant temporal decrease in prevalence (tau = −0.549; *p*-value = 0.02), while the main serovars remained stable. Network analysis revealed interconnections among serovars, exposure sources, and settings, with *S. typhimurium* acting as a central hub linking various animal hosts and exposure environments. Reptile-associated outbreaks were a significant subset, dominated by serovars *S*. Agbeni, *S*. Poona, *S*. Pomona, and *S*. Cotham.

**Conclusion:**

The findings of this study can inform public health authorities in their efforts to reduce the burden of NTS infections. The persistence of NTS serovars across diverse animal sources and exposure settings highlights the ongoing zoonotic transmission risk at the human-animal-environment interface. A comprehensive, One Health approach to prevention and control is suggested to mitigate the health burden of NTS outbreaks.

## 1 Introduction

Nontyphoidal *Salmonella enterica* (NTS) is a leading source of gastroenteritis in humans and pose a public health burden worldwide (1). Globally, NTS cause an estimated 153.1 million cases and 56,969 deaths (2). In the European Union (EU), 65,967 laboratory-confirmed NTS cases were reported in 2022, accounting for a case rate of 15.5 per 100,000 population (3). In the United States of America (U.S.), NTS is primarily transmitted through contaminated food of animal origin (mainly eggs, meat, poultry, and milk), with an estimated 1.3 million NTS foodborne illnesses, 12,500 hospitalizations, and 238 deaths occurring in 2019 (4). However, animal contact-related enteric diseases also contribute to the burden of salmonellosis. A recent U.S. enteric illness surveillance report documented 393 single-state and 164 multistate animal contact-related enteric disease outbreaks between 2009 and 2021, with NTS responsible for 52% of these outbreaks (5). The multitude of exposure sources and the transmission of NTS at the human animal-environment interface underscore the complexity and One Health characteristics of NTS (6–8). In addition, asymptomatic NTS colonization in livestock, poultry, and household pets can contribute to the persistent spread and maintenance of NTS, thus sustaining the human disease burden (6, 7). Moreover, a previous U.S. study compared foodborne and animal-contact-related NTS outbreaks and identified contact with backyard poultry and pet reptiles as a common source of animal-contact-related outbreaks, with young children, infants, and toddlers being more frequently represented (9).

A critical aspect of NTS epidemiology is the diversity among its serovars. To date, over 2,500 distinct serovars have been identified, each with various reservoirs, virulence factors, and ecological niches (6). Serovars *S. typhimurium*, *S. enteritidis*, and *S.* I 4,[5],12:i:- are the most prevalent NTS serovars that are responsible for zoonotic transmission (10). These serovars are often transmitted by poultry or poultry products (11, 12), pets (13), and livestock (14). Other non-common serovars include *S.* Agbeni, *S.* Cotham, *S.* Berta, *S.* Sandiego, *S.* Vitkin, *S.* Tennessee, and *S.* Chester, which are commonly transmitted through contact with reptiles and non-traditional pets (15, 16).

A previous study evaluated the enteric disease outbreak surveillance data reported to the Centers for Disease Control and Prevention (CDC) via the National Outbreak Reporting System (NORS) between 2009 to 2021, and identified serovar Typhimurium, I 4,[5],12:i:- as the main serovar among NTS outbreaks associated with animal contact (9). Despite the recognized role of animal contact in NTS infections, previous U.S. studies have not assessed serovar-specific epidemiological patterns, including the links between exposure sources, settings, and temporal trends in animal contact-related NTS outbreaks. Our study addresses this gap by examining serovar-specific source attribution and temporal patterns of NTS outbreaks, which can inform targeted intervention strategies. We hypothesize that the epidemiology of NTS animal contact-related outbreaks varies by serovar., with certain serovars linked to specific exposure sources and settings, and that temporal shifts may occur in the incidence of some serovars. To test this hypothesis, this study analyzes 14 years of NORS surveillance data (2009–2022) on single-state NTS outbreaks. This study aims to provide actionable data that will assist public health authorities in designing and implementing serovar., exposure source, and setting-specific prevention and control programs to reduce the health burden of NTS infections.

## 2 Materials and methods

### 2.1 Data source and study setting

The animal contact-related enteric outbreak data were obtained from the CDC through a data request. The dataset contained all animal-contact-related NTS outbreaks that were reported in NORS from 2009 to 2022. The data had details regarding NTS outbreak occurrence dates, geographic locations, exposure sources, and settings that were available in NORS as of August 2024. This study included NTS outbreaks where exposures occurred in a single state. The analysis was performed at the national level by integrating reported data from all jurisdictions. Population data for the study period were obtained from the U.S. Census Bureau (17).

### 2.2 Data analysis

#### 2.2.1 Descriptive statistics

The R software in the RStudio Platform (Version 1.4.1106, 2009–2021 RStudio, PBC) was employed for data cleaning and descriptive statistical analysis (18).

##### 2.2.1.1 Outbreak and illness incidence rates (IRs)

For each NTS serovar, the outbreak and illness IRs per 10 million person years (10MPY) for the study period (2009–2022) at the national level were calculated by using the following formulas:

$$\text{State}\;{\mathrm{IR}}_{i}\;\mathrm{per}\;10\mathrm{MPY}=\frac{\begin{array}{l} \frac{\begin{array}{l} \text{Number of outbreaks}\;\\ \text{in}\;Y1\;\text{in State}\;i \end{array}}{\text{Population in}\;Y1\;\text{in State}\;i}+\ldots \\ +\frac{\begin{array}{l} \text{Number of outbreaks}\;\\ \text{in}\;Y14\;\text{in State}\;i \end{array}}{\text{Population in}\;Y14\;\text{in State}\;i} \end{array}}{14}\times 10,000,000$$

$$\text{National}\;\mathrm{IR}\;\mathrm{per}\;10\;\mathrm{MPY}=\frac{\sum_{i}^{N}(\text{State}\;{\mathrm{IR}}_{i})}{N}$$

Where:

- N−the total number of states.
- $\text{State}\;{\mathrm{IR}}_{i}$ - the incidence rate per 10MPY for the state i

The illness incidence rate (IR) per 10MPY was calculated using the same formulas as for outbreaks, with the number of outbreaks replaced by the number of illnesses.

In addition, the number of outbreaks and illnesses for each serovar in each state was calculated.

##### 2.2.1.2 Serovars, exposure sources, and settings

For each serovar, the serovar proportion was calculated by using the following formula:


$$\text{Serovar Proportion}\;(\%)=\frac{\begin{array}{l} \text{Number of outbreaks}\;\\ \text{caused}\;\mathrm{by}\;\text{the serovar} \end{array}}{\begin{array}{l} \text{Total number}\;\\ \text{of outbreaks} \end{array}}\times 100$$

Serovar-specific proportions attributed to animal contact sources or settings were calculated by using the following formula:


$$\begin{array}{l} \text{Serovar}-\text{Specific Proportion of Outbreaks}\;\\ \text{Attributed to}\;a\;\text{Source or Setting}\;(\%)=\frac{\begin{array}{l} \text{Number of outbreaks}\;\\ \text{of}\;a\;\text{specific serovar}\;\\ \text{attributed to}\;a\;\\ \text{specific source}/\text{setting} \end{array}}{\begin{array}{l} \text{Total number of}\;\\ \text{outbreaks of}\;a\;\\ \text{specific serovar} \end{array}}\times 100 \end{array}$$

For all proportions, 95% confidence intervals (CIs) were calculated (19).

#### 2.2.2 Network analysis

To explore co-occurrences among the three most common serovars (Typhimurium, I 4,[5],12:i:-, and Enteritidis), exposure sources, and settings, a network analysis was conducted. The binary (presence = 1/ absence = 0) data were transformed into a co-occurrence matrix by multiplying the presence-absence matrix with its transpose (20), and variables with co-occurrence frequencies of more than 5% were retained. A weighted graph was generated using the igraph package in R (21). In the network, the nodes represented the specific serovars, exposure sources, and settings, while the edges depicted the strength of co-occurrence or association between them. Louvain community detection was also employed to identify clusters of related variables and key network matrices. For the network components, the degree, betweenness, clustering coefficients, and closeness factors were estimated (22, 23). The network was visualized with a directed layout.

##### 2.2.2.1 Multiple correspondence analysis (MCA)

To understand the co-occurrence patterns and potential relationships between serovars and exposure sources and settings, multiple correspondence analysis (MCA) was conducted (24). The MCA is a multivariate technique that reduces complex datasets to principal dimensions, facilitating pattern recognition and data visualization (25). Before the MCA analysis, categorical variables were assessed for variability, and those with a frequency of less than 5% were excluded from the analysis. Then, the selected variables were converted to a factor format, and a Burt matrix was constructed, which projected the data into a reduced-dimensional space. At the last step, the first two dimensions, which accounted for the greatest proportion of total inertia (variability), were illustrated in two-dimensional MCA plots.

The MCA included the three most common serovars (*S. typhimurium*, *S. enteritidis*, and *S*. I 4,[5],12:i-), the exposure source categories (bird, mammal, reptile, and NA) and the exposure settings (farm/dairy/agricultural; child daycare/preschool; school/college/university; correctional/detention facility; animal shelter or sanctuary; agricultural feed store; veterinary clinic; residence—single-family home; residence—multi-unit housing; unknown, and NA).

##### 2.2.2.2 Trend analysis

Temporal trends in serovar proportions were assessed using Locally Estimated/Weighted Scatterplot Smoothing (LOESS) and the Mann–Kendall (MK) test (26–28). LOESS was used to visualize smoothed temporal patterns without assuming a specific distribution, linearity, or significance. At the same time, the MK test was applied to assess the presence, direction, and statistical significance of monotonic trends over time.

## 3 Results

### 3.1 Outbreak and illness incidence rates

The national-level animal contact-related NTS outbreak and illness IRs per 10 million person years (10MPY), as well as the mean number of illnesses per outbreak, are presented in Table 1.

**Table 1:** Serovar-specific nontyphoidal *Salmonella enterica* outbreak and illness incidence rates, and the mean number of illnesses per outbreak, United States, 2009–2022.

Serovar	Outbreaks	Outbreak IRs per 10MPY	Illnesses	Illness IRs per 10MPY	Illnesses per outbreak
Agbeni	1	0.60	14	7.80	14.00
Berta	1	1.60	2	3.30	2.00
Braenderup	4	1.50	8	3.00	2.00
Cotham	1	1.30	2	2.60	2.00
Durban	1	5.00	2	10.10	2.00
Enteritidis	10	3.70	39	18.50	3.90
Gaminara	1	2.50	2	4.90	2.00
Hadar	2	2.10	6	6.70	3.00
Hartford	1	2.30	1	2.30	1.00
Havana	1	1.20	3	3.70	3.00
Heidelberg	2	2.70	3	3.30	1.50
I 4,[5],12:i:-	15	5.20	67	17.70	4.47
Infantis	1	2.30	5	11.50	5.00
Javiana	1	0.90	3	2.60	3.00
Johannesburg	1	0.00	7	0.01	7.00
Lomalinda	1	2.20	2	4.50	2.00
Mbandaka	1	3.70	2	7.40	2.00
Montevideo	4	1.20	20	6.70	5.00
Muenchen	1	3.70	2	7.40	2.00
Newport	1	2.40	1	2.40	1.00
Paratyphi B var. L(+) tartrate +	1	1.20	2	2.40	2.00
Pomona	3	7.70	5	15.20	1.67
Poona	1	0.60	2	1.10	2.00
Saintpaul	3	3.50	19	28.60	6.33
Sandiego	1	0.70	4	2.90	4.00
Stanley	1	4.10	2	8.20	2.00
Telelkebir	1	1.60	3	4.90	3.00
Thompson	2	0.00	29	0.01	14.50
Typhimurium	33	3.70	186	25.20	5.63
Uganda	1	1.30	2	2.60	2.00
Unknown	3	1.90	2	1.50	0.67

The serovars with the highest IR per 10MPY were: Pomona and I 4,[5],12:i:-, while the serovars with the highest illness IRs were Saintpaul, Typhimurium, Enteritidis, and I 4,[5],12:i:-.

The state-level outbreak and illness numbers varied by serovar and state (Table 2).

**Table 2:** Serovar-specific nontyphoidal *Salmonella enterica* outbreaks and illnesses by states, United States, 2009–2022.

Serovar	State	Outbreak	Illness
Agbeni	Pennsylvania	1	14
Berta	Kentucky	1	2
Braenderup	Ohio	1	2
Braenderup	Texas	1	2
Braenderup	West Virginia	1	2
Braenderup	Wisconsin	1	2
Cotham	Colorado	1	2
Durban	Hawaii	1	2
Enteritidis	Alabama	1	5
Enteritidis	Colorado	1	2
Enteritidis	Minnesota	1	2
Enteritidis	New Hampshire	1	9
Enteritidis	Pennsylvania	1	2
Enteritidis	South Carolina	1	3
Enteritidis	Tennessee	1	4
Enteritidis	Vermont	1	2
Enteritidis	Virginia	1	3
Enteritidis	Wyoming	1	7
Gaminara	Kansas	1	2
Hadar	Oregon	1	2
Hadar	Utah	1	4
Hartford	Iowa	1	1
Havana	Wisconsin	1	3
Heidelberg	Idaho	1	1
Heidelberg	Minnesota	1	2
I 4,[5],12:i:-	Arizona	1	6
I 4,[5],12:i:-	Colorado	1	20
I 4,[5],12:i:-	Idaho	1	1
I 4,[5],12:i:-	Illinois	1	3
I 4,[5],12:i:-	Michigan	1	4
I 4,[5],12:i:-	Minnesota	1	4
I 4,[5],12:i:-	Nebraska	1	0
I 4,[5],12:i:-	Ohio	2	7
I 4,[5],12:i:-	Utah	2	6
I 4,[5],12:i:-	Wisconsin	1	6
I 4,[5],12:i:-	Wyoming	3	10
Infantis	Iowa	1	5
Javiana	Virginia	1	3
Johannesburg	NA	1	7
Lomalinda	Utah	1	2
Mbandaka	Nebraska	1	2
Montevideo	Colorado	1	4
Montevideo	Oregon	1	6
Montevideo	Pennsylvania	1	1
Montevideo	Washington	1	9
Muenchen	Idaho	1	2
Newport	Arkansas	1	1
Paratyphi B var. L(+) tartrate +	Missouri	1	2
Pomona	Alaska	1	2
Pomona	Ohio	1	1
Pomona	Wyoming	1	2
Poona	Pennsylvania	1	2
Saintpaul	Massachusetts	1	3
Saintpaul	Pennsylvania	1	7
Saintpaul	South Dakota	1	9
Sandiego	Michigan	1	4
Stanley	Idaho	1	2
Telelkebir	Kentucky	1	3
Thompson	NA	2	29
Typhimurium	Arizona	1	0
Typhimurium	Colorado	3	19
Typhimurium	Indiana	1	2
Typhimurium	Iowa	1	12
Typhimurium	Maryland	1	5
Typhimurium	Michigan	3	22
Typhimurium	Minnesota	2	5
Typhimurium	Montana	1	3
Typhimurium	New Hampshire	2	39
Typhimurium	New Mexico	1	7
Typhimurium	New York	1	2
Typhimurium	North Carolina	1	29
Typhimurium	Ohio	6	9
Typhimurium	Oregon	2	6
Typhimurium	Utah	4	21
Typhimurium	Vermont	1	2
Typhimurium	Virginia	1	0
Typhimurium	Wisconsin	1	3
Uganda	Minnesota	1	2

The highest number of outbreaks was identified in Ohio (*n* = 12), and these outbreaks were linked to serovars Braenderup (*n* = 1), I 4,[5],12:i:- (*n* = 2), Pomona (*n* = 1), and Typhimurium (*n* = 6). Additionally, Utah reported 8 outbreaks linked to serovars Hadar (*n* = 1), I 4,[5],12:i:- (*n* = 2), Lomalinda (*n* = 1), and Typhimurium (*n* = 4); Colorado reported 7 outbreaks linked to serovars Cotham, Enteritidis, I 4,[5],12:i:-, Montevideo (1 outbreak each), and Typhimurium (n = 3); Minnesota reported 6 outbreaks linked to serovars Enteritidis, Heidelberg, I 4,[5],12:i:-, and Uganda (1 outbreak each), and Typhimurium (*n* = 2) (Table 2).

### 3.2 Proportion of serovars

Among the 104 reported animal-contact-related NTS outbreaks, 32 different serovars were detected. The highest number of outbreaks was linked to *S. typhimurium* (*n* = 33; 32.67%; 95% CI: 24.57–42.31), followed by *S.* I 4,[5],12:i:- (*n* = 15; 14.85%; 95% CI: 9.21–23.07), and *S. enteritidis* (*n* = 10; 9.90%; 95% CI: 5.31–16.80). Four outbreaks each (3.96%; 95% CI: 1.55–9.74) were linked to serovars Braenderup and Montevideo, three outbreaks each (2.97%; 95% CI: 1.02–8.37) to serovars Pomona and Saintpaul, two outbreaks each (1.98%; 95% CI: 0.54–6.93) to serovars Hadar, Heidelberg, and Thompson, and one outbreak each (0.99, 95% CI: 0.17–5.40) to serovars Agbeni, Berta, Cotham, Durban, Gaminara, Hartford, Havana, Infantis, Javiana, Johannesburg, Lomalinda, Mbandaka. Muenchen, Newport, Paratyphi B var. L(+) tartrate +, Poon, Sandiego, Stanley, Telelkebir, and Uganda. For two outbreaks, no serovar information was reported. For three outbreaks, serovars were either misspelled or misclassified and were excluded from calculations.

### 3.3 Proportions of exposure sources and settings

**Figure 1:**
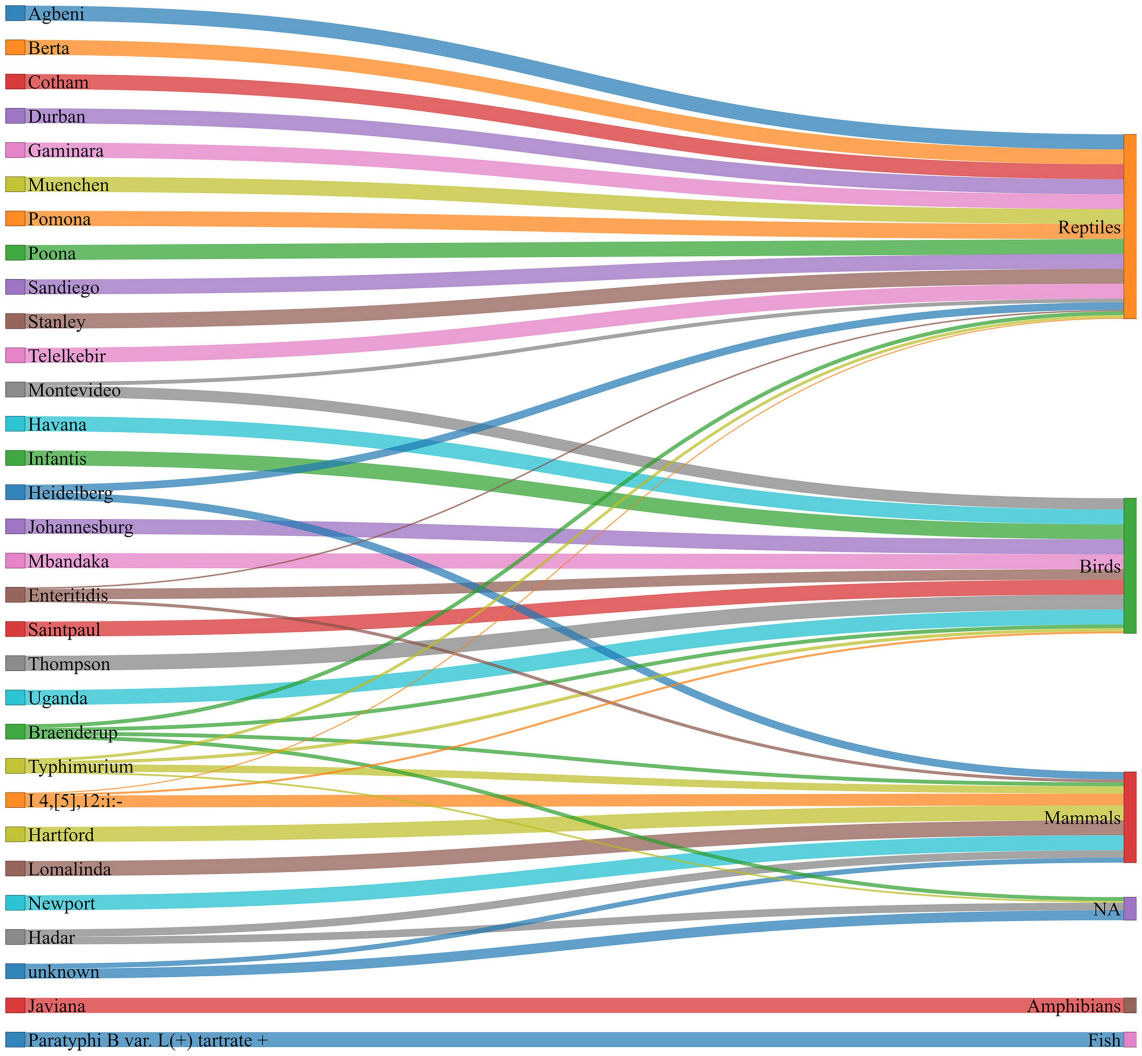
Distribution of animal contact-related outbreaks by serovars and animal source categories, U.S., 2009–2022. The bands (different colors) and the bandwidth indicate the strength of association between a serovar and exposure source category. The categories and subcategories of exposure sources are: amphibians (frogs), birds (poultry [baby chick, duckling, chicken, ducks, turkey, backyard poultry, and other poultry], vulture, and songbirds), fish (pet fish), mammals (horse, cattle, calf, baby cow, sheep, pig, dog, hedgehog, ferret, guinea pig, and mouse), reptiles (snakes, turtle, lizard [bearded dragon, and leopard gecko], other reptiles, and NA [no information available]).

Animal contact exposure sources were associated with a diverse range of serovars, each linked to different sources (Figure 1).

Among the 33 NTS outbreaks linked to serovar Typhimurium, 16 outbreaks (48.5%) were linked to mammals, including cattle, dogs, horses, pigs, guinea pigs, hedgehogs, and mice; 7 outbreaks (21.2%) were linked to birds, including baby chicks, ducklings, and other poultry; 6 outbreaks (18.2%) were linked to reptiles, including lizards, snakes, and turtles; and 4 outbreaks (12.1%) had unknown exposure source (Figure 1, Supplementary Table S1).

Among the 15 NTS outbreaks associated with serovar I 4,[5],12:i:-, 12 outbreaks (80%) were linked with mammals (cattle, pig, and sheep), 2 outbreaks (13.3%) with birds (chickens and Turkeys), and 1 outbreak (6.7%) with reptiles (turtles).

Among the 10 NTS outbreaks linked with serovar Enteritidis, 7 outbreaks (70%) were linked with birds (baby chicks, ducklings, Buff Orpington chickens, and vultures), 2 outbreaks (20%) with mammals (ferrets and mice), and 1 outbreak (10%) with reptiles (turtle). Among the 4 NTS outbreaks associated with serovar Braenderup, one outbreak each (25%) was linked to birds (baby chicks), mammals (guinea pig), reptiles (small turtle and Lizard), and NA (not available). Among the 4 NTS outbreaks linked to serovar Montevideo, three outbreaks (75%) were linked with birds (baby chicks and ducklings), and one outbreak (25%) with reptiles (turtles). Serovars Pomona and Poona serovars were entirely (100%) associated with reptiles (Red-eared slider turtle), while serovar Saintpaul had 100% of outbreaks linked with birds (chickens and ducklings). (Figure 1 and Supplementary Table S1).

The less frequent serovars, including *S.* Hadar, *S.* Heidelberg, *S. paratyphi* B var. L(+) tartrate +, *S.* Javiana, *S.* Johannesburg, and *S.* Mbandaka were linked to various single animal exposure sources (Supplemetnary Table S1).

**Figure 2:**
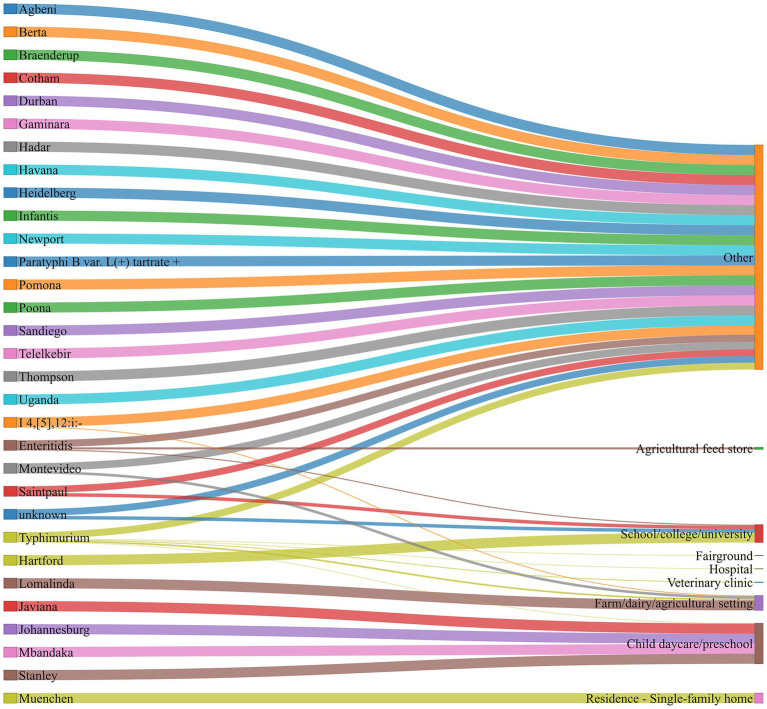
Distribution of animal contact-related outbreaks by serovars and exposure settings, U.S., 2009–2022. The bands (different colors) and the bandwidth indicate the strength of association between a serovar and an exposure setting.

Among the animal contact-associated NTS outbreaks, exposure settings were associated with a diverse range of serovars, each linked to different settings (Figure 2, Supplementary Table S1).

### 3.4 Network analysis

**Figure 3:**
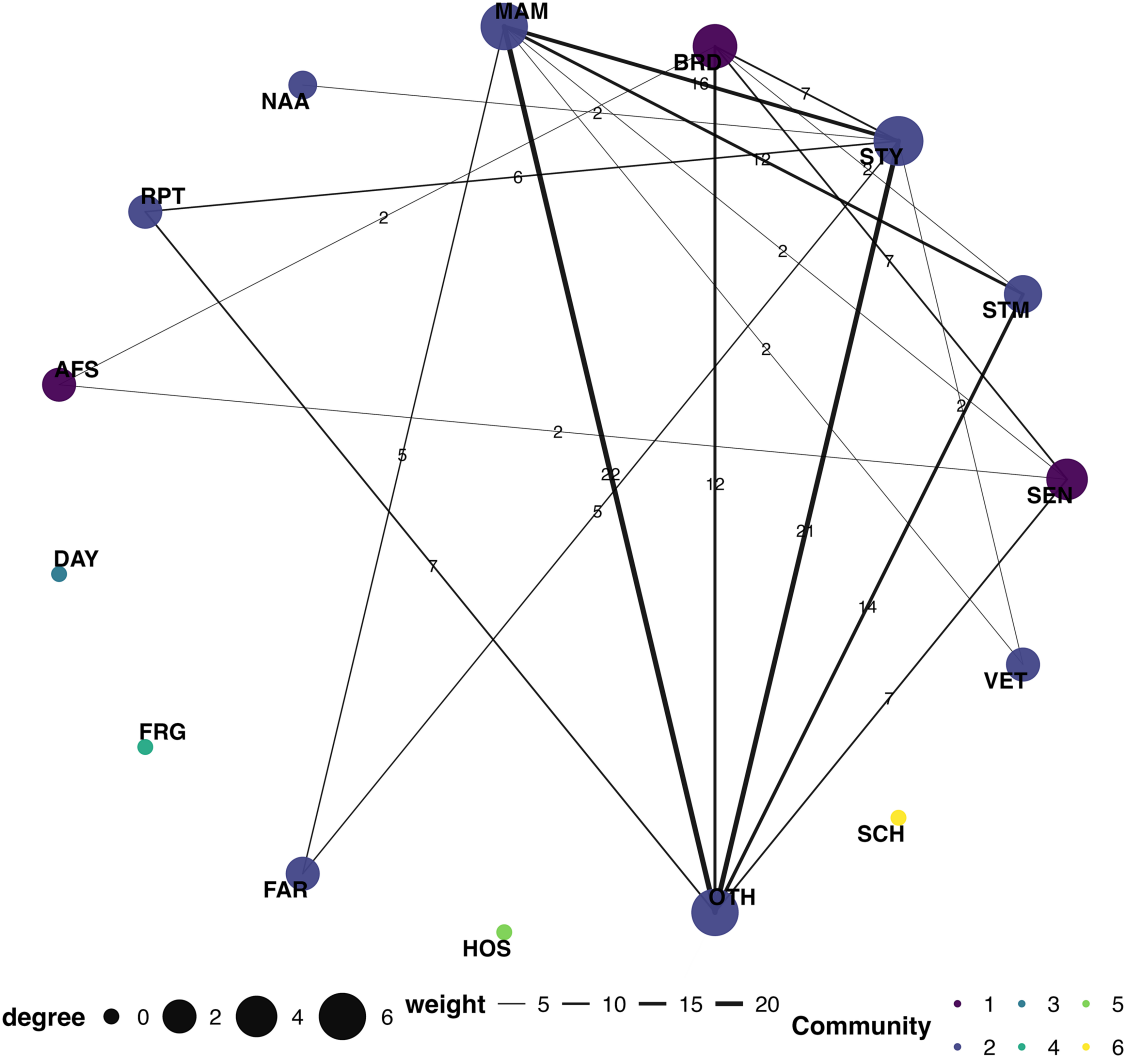
Co-occurrence network of serovars, exposure sources, and settings, U.S., 2009–2022. SEN (*S. enteritidis*), STM (*S.* I 4,[5],12:i:-), STY (*S. typhimurium*), BRD (Birds), MAM (Mammals), NAA (Not available), RPT (Reptiles), AFS (Agricultural feed stores), DAY (Child daycare/preschool), FRG (Fairgrounds), FAR (Farm/dairy/agricultural setting), HOS (Hospital), OTH (Others), SCH (School/college/university), and VET (Veterinary clinics).

A network analysis was conducted that included serovars Typhimurium, Enteritidis, and I 4,[5],12:i:-, along with the exposure sources and exposure settings (Figure 3).

The final network consisted of fifteen nodes and seventeen edges after applying a co-occurrence threshold of five. Each node represented either a serovar., an exposure source, or an exposure setting, while edges indicated the frequency of co-occurrence among these variables. Louvain community detection identified nine distinct modular communities within the network, showing varying levels of interconnectivity. Serovar Enteritidis clustered closely with birds, forming community 1. Serovars Typhimurium and I 4,[5],12:i:- were part of community 2, together with mammals, reptiles, farm/dairy/agricultural settings, and Other exposure settings, by forming the most extensive and interconnected cluster. The remaining nodes, unknown exposure source, agricultural feed stores, child daycare/preschool, fairground, hospital, school/college/university, and veterinary clinic, each formed smaller or isolated communities 3–9, indicating weak or no direct co-occurrence relationships with the important serovars or animal contact categories (Figure 3, Supplementary Table S2).

The network centrality measures showed variations in connectivity and structural influence across the nodes. The node representing serovar Typhimurium had a degree of 5 and the highest betweenness of 8, highlighting its central importance in bridging multiple nodes. Mammals had degree values of 2 and betweenness of 0, reflecting limited but direct connections. Reptiles and farm/dairy/agricultural settings also showed moderate connections, with the degree values of 2 and betweenness centrality of 2 and 5, respectively. Nodes with an unknown animal source category, agricultural feed store, fairground, child daycare/preschool, hospital, school/college/university, and veterinary clinic had degree values of 0, reflecting complete isolation. The node representing other exposure settings exhibited the highest degree of 6 and moderate betweenness centrality of 4, indicating a stronger role as a connector in the network (Figure 3, Supplementary Table S2).

The closeness centrality of nodes ranged between 0.075 and 0.0129, with *S. typhimurium* showing the highest values of 0.012, followed by exposure source, representing reptiles (0.011), and farm/dairy/agricultural settings (0.011), indicating their proximity to other nodes in the network. Serovar I 4,[5],12:i:- showed the lowest closeness values of 0.008. The clustering coefficients ranged between 0.33 and 1.00, with the highest values of 1.00 for serovars Enteritidis and I 4,[5],12:i:-, and reptiles, and farm/dairy/agricultural settings, signifying tightly connected communities. Birds (0.67) and mammals (0.50) also showed relatively higher clustering coefficients. Whereas other exposure settings showed the lowest value of 0.33, indicating a bridging among groups with lesser similarity (Figure 3, Supplementary Table S2).

### 3.5 Multiple correspondence analysis (MCA)

**Figure 4:**
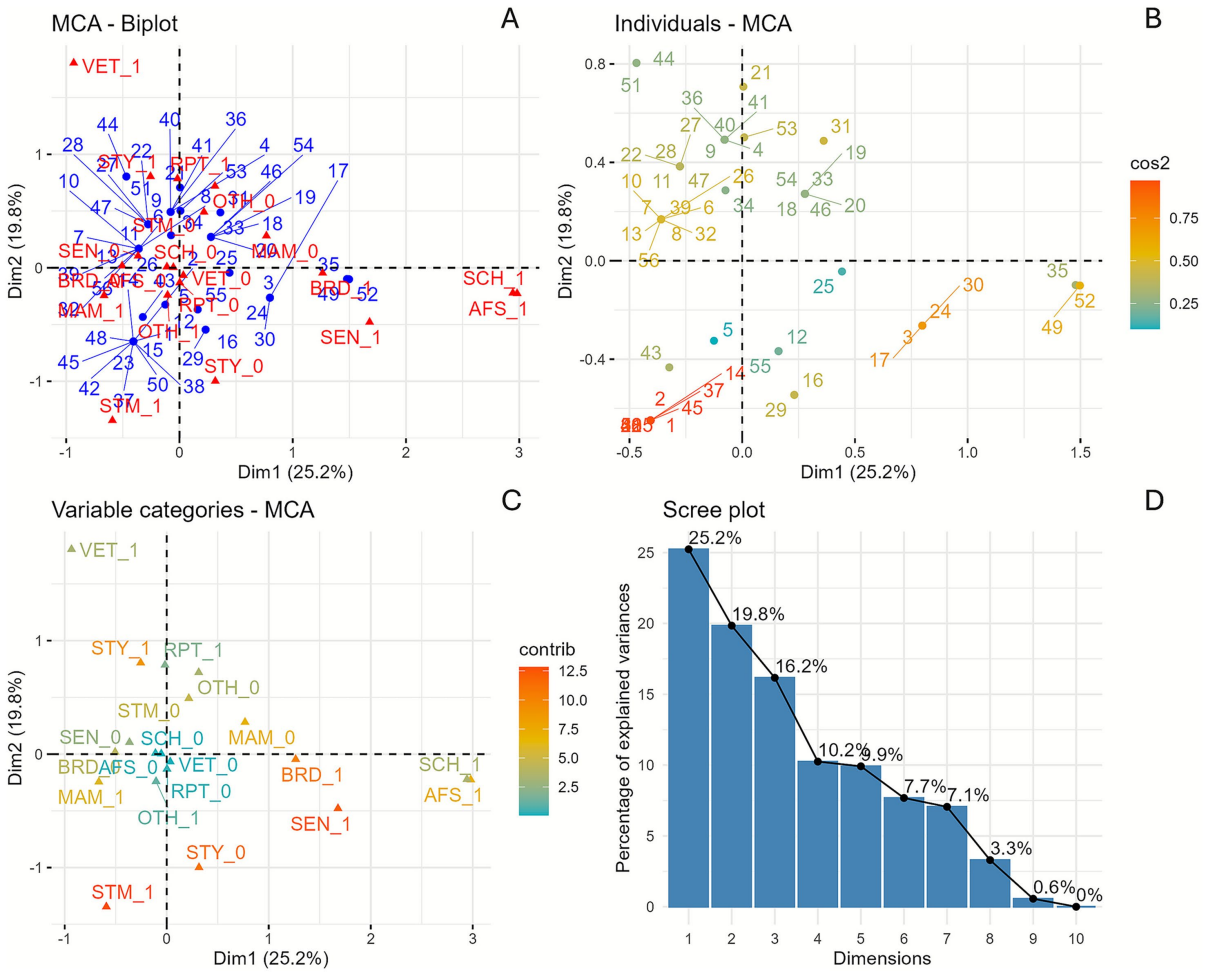
Multiple correspondence analysis of the three most common *Salmonella enterica* serovars and associated exposure sources and settings, U. S. 2009–2022. **(A)** MCA biplot showing the distribution of outbreak reports, serovars, exposure sources, and settings. **(B)** The individual contribution plot reveals the relative influence of each outbreak on the total variance. **(C)** The variable categories plot reflects the influence of each serovar., exposure source, and setting. **(D)** Scree-plot depicts the eigenvalue and percentage of explained variance in each dimension. SEN (*S. enteritidis*), STM (*S.* I 4,[5],12:i:-), STY (*S. typhimurium*), BRD (Birds), MAM (Mammals), NAA (Not available), RPT (Reptiles), AFS (Agricultural feed stores), DAY (Child daycare/preschool), FRG (Fairgrounds), FAR (Farm/dairy/agricultural setting), HOS (Hospital), OTH (Others), SCH (School/college/university), and VET (Veterinary clinics).

The MCA included the three most common serovars (*S. typhimurium*, *S. enteritidis*, and *S.* I 4,[5],12:i:-) and the exposure source categories and exposure settings, which were illustrated in Figure 4, and described in Supplementary Tables S3–S5.

The first two dimensions explained 45% of the total inertia (variance), with dimensions 1 (25.2%) and dimension 2 (19.8%), respectively. The most prominent contributors to the variance in dimension 1 were the presence of serovar Enteritidis (19.94%) (SEN_1) and exposure source birds (18.09%) (BRD_1), both of which were closely located on the right side of the two-dimensional plot (Figure 4C), suggesting frequent co-occurrence. Dimension 2 was primarily influenced by serovar I 4,[5],12:i:- (24.42%) (STM_1) and veterinary clinics (VET_1); however, they were located at opposite ends of the graph, suggesting that they did not co-occur (Figure 4C). Other contributors to dimension 2 included serovar Typhimurium (18.08%) (STY_1) and reptiles (4.44%) (RPT_1), which were closely located at the top-center of the two-dimensional plot, suggesting frequent co-occurrence.

The MCA biplot confirmed that the *S. enteritidis*, the agricultural feed store, and the birds showed the longest vectors in dimension 1, indicating the highest contribution to this dimension. In dimension 2 serovars I 4,[5],12:i:-, and Typhimurium were more pronounced (Figure 4A). The cos^2^ plot (Figure 4B) showed that outbreaks 1, 2, 10, and 13 were the most strongly represented in MCA space (cos^2^ > 0.75), indicating stronger alignment with dimensions.

### 3.6 Temporal trend analysis

**Figure 5:**
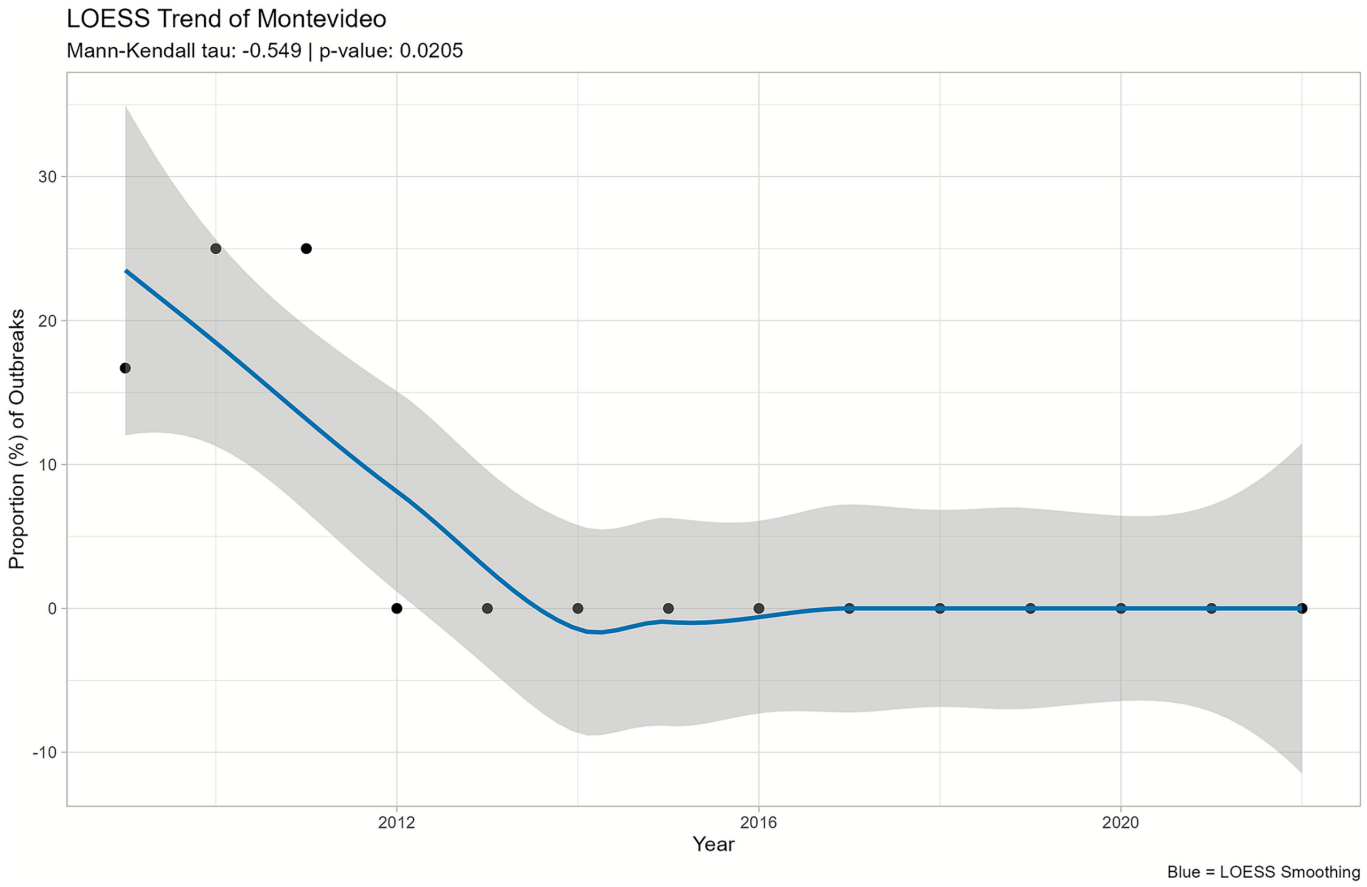
Trend analysis of *Salmonella enterica* serovar Montevideo proportion in animal contact-related outbreaks, U.S., 2009–2022. The LOESS plot curve depicts changes in the serovar outbreak proportion over time, while the shaded area on the plot reflects the 95% confidence interval.

Among all the serovars analyzed, only *S*. Montevideo demonstrated a statistically significant decreasing monotonic trend (tau = −0.549; *p*-value = 0.02) over the 14-year study period (Figure 5).

All the other serovars exhibited nonsignificant MK test results. Their LOESS curves reflected stable or fluctuating patterns over time without consistent increasing or decreasing trends (Supplementary Figures S1, S2).

## 4 Discussion

This study analyzed the serovar-level animal contact-related NTS single-state outbreak data reported between 2009 and 2022 to the CDC through NORS by state, local, and territorial public health agencies across the U.S. A total of 32 distinct serovars were associated with 104 different outbreaks. The top three serovars linked to animal contact outbreaks were Typhimurium, I 4,[5],12:i:-, and Enteritidis. Co-occurrences were identified between these serovars and their exposure sources and settings. Serovar Enteritidis was linked with poultry, while serovars Typhimurium and I 4,[5],12:i:- were associated with mammals, reptiles, and farm/dairy/agricultural settings. Among the 32 serovars, only serovar Montevideo, a less common serovar., showed a significant temporal decrease in its prevalence. On the other hand, the main serovars, Typhimurium, I 4,[5],12:i:-, and Enteritidis trends remained stable over time, suggesting that they are consistently present in animal sources and pose a continuous health burden.

In this study, serovar Typhimurium was the most common serovar associated with animal-contact-related outbreaks, which exhibited consistently high incidence rates of both outbreaks and illnesses. This finding can be attributed to its broad ecological adaptability, which allows serovar Typhimurium to thrive in multiple animal hosts. Its presence in cattle, pigs, poultry, household pets, reptiles, and songbirds continues to pose a significant zoonotic transmission risk (29–32).

The second most common serovar in our study was serovar I 4,[5],12:i:-. A previous study has described serovar I 4,[5],12:i:- in human outbreaks, with 63% of these associated with a multidrug-resistant clade linked to pork consumption or contact with swine (33). Although serovar I 4,[5],12:i:- is primarily linked to swine (34), other animals, including chickens, bovine, and turkeys, have also been described as sources (35).

The third most common serovar in this study, *S. enteritidis*, showed strong associations with birds and feed stores in the MCA and network analysis. Previous studies have confirmed this finding by highlighting the frequent zoonotic transmission of *S. enteritidis* via contact with live poultry in agricultural feed stores (12).

In our study, serovar Enteritidis showed a strong link to bird reservoirs, including backyard poultry (such as baby chicks, ducklings, chickens, ducks, and turkeys) and non-poultry bird species (vultures), emphasizing a consistent public health concern. This finding is consistent with previous studies from the U.S., identifying serovar Enteritidis as a common source for commercial and backyard poultry contact-related outbreaks (36).

Reptile-associated NTS outbreaks constituted a significant subset of animal contact-related outbreaks in our study, with serovars such as *S*. Agbeni, *S*. Poona, *S*. Pomona, and *S*. Cotham being predominant. These NTS serovars have previously been reported in both single- and multistate reptile and amphibian-associated outbreaks in the U.S. (37) and Canada (16). Exposure to reptiles constitutes a zoonotic transmission risk, and an education campaign raising awareness, especially among children, is needed to mitigate the transmission of these serovars to humans (16, 37).

The network analysis in our study revealed interconnections among different nontyphoidal *Salmonella enterica* serovars, exposure source categories, and exposure settings, highlighting relationships within the NTS outbreaks. Interestingly, *Salmonella Typhimurium* was identified as the central hub in the network, linking a variety of mammalian hosts, birds, reptiles, agricultural and farm environments, and other exposure settings. This centrality suggests that *S. typhimurium* might serve as an important serovar able to bridge multiple exposure sources and settings, facilitating its transmission across diverse environments and host species. This finding suggests that a broader network of transmission pathways should be considered when developing control strategies, as pathogens like *S. typhimurium* can traverse multiple pathways to maintain persistence in both animal populations, posing a constant human health risk.

While serovar I 4,[5],12:i:- had a bridging position between mammal and reptile nodes, which suggests that this serovar could be present in multiple host species. This finding was also described in a previous study showing a flexible host adaptation and multiple exposure settings for this serovar (38).

In our study, serovar Montevideo had a high prevalence at the beginning of the study period, being implicated in 3 outbreaks, but after 2011, no outbreaks were linked to this serovar. A previous U. S. study examining live poultry-associated outbreaks between 1990 and 2014 found that serovar Montevideo was identified in 36% (19/53) of these outbreaks, making it the most commonly reported serovar (42). However, based on our results, it seems that this serovar in the poultry flocks was replaced by other serovars, such as serovar Enteritidis.

Our serovar-level approach revealed that a small number of serovars, primarily *S. typhimurium*, *S. enteritidis*, and *S.* I 4,[5],12:i:-, accounted for most of NTS outbreaks, while various rare NTS serovars emerged periodically, and were linked to specific animal sources such as exotic pets, reptiles, and wildlife. Such outbreak patterns suggest a multifaceted NTS outbreak epidemiology, in which persistent and emerging serovars coexist across multiple ecological niches, shaped by complex human, animal, and environmental interactions (39, 40).

Beyond the commonly detected NTS serovars, several less frequently identified serovars exhibited distinct exposure settings, adding complexity to the transmission dynamics of zoonotic NTS. Reptile-associated serovars, including *S*. Vitkin, *S*. Pomona, *S*. Poona, *S*. Cotham, *S*. Agbeni, and *S*. Berta, were exclusively linked to household exposure settings, highlighting the specific transmission routes and risk factors associated with these serovars. This finding agrees with previous Canadian studies describing reptile-associated human NTS outbreaks (16, 41).

As all studies have limitations, our study is no exception. Our study focused on local NTS outbreaks that have exposures in a single state, and future studies should assess multistate NTS outbreaks to identify novel exposures and settings. In addition, our study assessed NTS outbreaks voluntarily reported to the CDC by local public health authorities, and differences in reporting, underreporting, incomplete serovar., exposure source, and settings documentation might have occurred that could affect our study results.

## 5 Conclusion

Our study provides new serovar-specific information on exposure sources and settings of NTS outbreaks linked to animal contact across the United States. The dominance of main serovars, such as *S. typhimurium*, *S*. I 4,[5],12:i:-, and *S. enteritidis*, suggests their adaptability and persistence across diverse animal sources and exposure settings, making it challenging to mitigate their transmission risk at the human-animal-environment interface. Additionally, several less frequently observed serovars, including *S*. Vitkin, *S*. Pomona, and *S*. Poona, were exclusively linked to reptile contact. These findings can aid public health authorities in developing integrated prevention and control strategies to reduce the burden of NTS infections.

## Data Availability

The original contributions presented in the study are included in the article/Supplementary material, further inquiries can be directed to the corresponding author.
