# Supplementary material for: Animal contact-related nontyphoidal Salmonella enterica outbreaks in humans in the U.S. (2009–2022): serovar-specific temporal trends and associations with exposure sources and settings
Source: Front Public Health. 2026 Feb 4;14:1755882. doi: 10.3389/fpubh.2026.1755882 (PMC12913571; doi:10.3389/fpubh.2026.1755882)
Supplement: Supplementary file 1 [file Supplementary_file_1.docx]

**Supplementary Information**

**Supplementary Tables**

**Table S1. Nontyphoidal *Salmonella enterica* serovar outbreaks associated with animal contact categories and exposure settings proportions across the U.S. 2009 – 2022.**

| **Serovars** | **Animal category** | **Animal types** | **Exposure settings** | **Total Outbreaks** | **Count** | **Proportion (%)** | **95% Confidence Interval** |
| --- | --- | --- | --- | --- | --- | --- | --- |
| Agbeni | Reptiles | Turtle | Other | 1 | 1 | 100 | 20.7 - 100 |
| Berta | Reptiles | Turtle - Turtle | Other | 1 | 1 | 100 | 20.7 - 100 |
| Braenderup | Birds | Baby chick or duckling - duckling | Other | 4 | 1 | 25 | 4.6 - 69.9 |
| Braenderup | Mammals | Other small mammalian household pet - Guinea pig | Other | 4 | 1 | 25 | 4.6 - 69.9 |
| Braenderup | NA | NA |  | 4 | 1 | 25 | 4.6 - 69.9 |
| Braenderup | Reptiles | Lizard - Gecko | Other | 4 | 1 | 25 | 4.6 - 69.9 |
| Cotham | Reptiles | Lizard - Bearded dragon | Other | 1 | 1 | 100 | 20.7 - 100 |
| Durban | Reptiles | Lizard - Jackson's chameleon | Other | 1 | 1 | 100 | 20.7 - 100 |
| Enteritidis | Birds | Baby chick or duckling, Baby chick or duckling - Baby chicks, Baby chick or duckling - Buff Orpingtons, Baby chick or duckling - Chick, baby chick, Bird, not including poultry - Vulture | Agricultural feed store, Other, School/college/university | 10 | 7 | 70 | 39.7 - 89.2 |
| Enteritidis | Mammals | Other small mammalian household pet - Ferret, Other small mammalian household pet - Mouse | Other | 10 | 2 | 20 | 5.7 - 51 |
| Enteritidis | Reptiles | Turtle - Turtle | Other | 10 | 1 | 10 | 1.8 - 40.4 |
| Gaminara | Reptiles | Lizard - Bearded dragon | Other | 1 | 1 | 100 | 20.7 - 100 |
| Hadar | Mammals | Baby chick or duckling - Chick, baby chick | Other | 2 | 1 | 50 | 9.5 - 90.5 |
| Hadar | NA | NA |  | 2 | 1 | 50 | 9.5 - 90.5 |
| Hartford | Mammals | Other (specify) - Horse | School/college/university | 1 | 1 | 100 | 20.7 - 100 |
| Havana | Birds | Other poultry - Turkey | Other | 1 | 1 | 100 | 20.7 - 100 |
| Heidelberg | Mammals | Cattle - Calves | Other | 2 | 1 | 50 | 9.5 - 90.5 |
| Heidelberg | Reptiles | Other reptile - Black roughneck monitor lizard | Other | 2 | 1 | 50 | 9.5 - 90.5 |
| I 4,[5],12:i:- | Birds | Other poultry - Chicken, Other poultry - Turkey | Other | 15 | 2 | 13.3 | 3.7 - 37.9 |
| I 4,[5],12:i:- | Mammals | Cattle, Cattle - Cattle and Goats: Custom Slaughter workers, Other (specify) - cattle, sheep or goats, Pig, Pig - Hog, Pig - Pig/hog (domestic), Pig - Pigs, Sheep or goats - Sheep | Farm/dairy/agricultural setting, Other | 15 | 12 | 80 | 54.8 - 93 |
| I 4,[5],12:i:- | Reptiles | Turtle - Turtle | Other | 15 | 1 | 6.7 | 1.2 - 29.8 |
| Infantis | Birds | Baby chick or duckling - Chick, baby chick | Other | 1 | 1 | 100 | 20.7 - 100 |
| Javiana | Amphibians | Amphibian - Frogs | Child daycare/preschool | 1 | 1 | 100 | 20.7 - 100 |
| Johannesburg | Birds | Baby chick or duckling - Chick, baby chick | Child daycare/preschool | 1 | 1 | 100 | 20.7 - 100 |
| Lomalinda | Mammals | Cattle - baby cow | Farm/dairy/agricultural setting | 1 | 1 | 100 | 20.7 - 100 |
| Mbandaka | Birds | Baby chick or duckling - Chicks | Child daycare/preschool | 1 | 1 | 100 | 20.7 - 100 |
| Montevideo | Birds | Baby chick or duckling - Chick, baby chick | Farm/dairy/agricultural setting, Other | 4 | 3 | 75 | 30.1 - 95.4 |
| Montevideo | Reptiles | Turtle - Turtle | Other | 4 | 1 | 25 | 4.6 - 69.9 |
| Muenchen | Reptiles | Lizard, bearded dragon | Residence - Single-family home | 1 | 1 | 100 | 20.7 - 100 |
| Newport | Mammals | Other small mammalian household pet - Ferret | Other | 1 | 1 | 100 | 20.7 - 100 |
| Paratyphi B | Fish | Pet fish - Fish | Other | 2 | 2 | 100 | 34.2 - 100 |
| Paratyphi B var. L(+) tartrate + | Fish | Pet fish - Fish | Other | 1 | 1 | 100 | 20.7 - 100 |
| Pomona | Reptiles | Other reptile - Reptile, other, Turtle - Turtle | Other | 3 | 3 | 100 | 43.9 - 100 |
| Poona | Reptiles | Turtle - Red-eared slider turtle | Other | 1 | 1 | 100 | 20.7 - 100 |
| Saintpaul | Birds | Baby chick or duckling - ducklings, Other poultry - Chicken | Other, School/college/university | 3 | 3 | 100 | 43.9 - 100 |
| Sandiego | Reptiles | Turtle - Turtle | Other | 1 | 1 | 100 | 20.7 - 100 |
| Stanley | Reptiles | Other reptile - Corn snake | Child daycare/preschool | 1 | 1 | 100 | 20.7 - 100 |
| Telelkebir | Reptiles | Lizard - Bearded dragon | Other | 1 | 1 | 100 | 20.7 - 100 |
| Thompson | Birds | Baby chick or duckling - Chick, baby chick | Other | 2 | 2 | 100 | 34.2 - 100 |
| Typhi | Birds | Baby chick or duckling - Duck | Other | 1 | 1 | 100 | 20.7 - 100 |
| Typhimurium | Birds | Baby chick or duckling - Chick, baby chick, Other poultry - Chicken, Other poultry - duckling, Other poultry - Poultry, other | Farm/dairy/agricultural setting, Other | 33 | 7 | 21.2 | 10.7 - 37.8 |
| Typhimurium | Mammals | Cattle, Cattle - Cattle, Cattle - Cattle, calf, Cattle - Holstein and Jersey, Dog or Puppy - Dog, Dog or Puppy - Dog, puppy, Other (specify) - Horse, Other (specify) - Livestock, other, Other small mammalian household pet - Guinea pig, Other small mammalian household pet - Hedgehog, Other small mammalian household pet - Mouse | Farm/dairy/agricultural setting, Hospital, Other, Veterinary clinic | 33 | 16 | 48.5 | 32.5 - 64.8 |
| Typhimurium | NA | NA | Fairground, Other | 33 | 4 | 12.1 | 4.8 - 27.3 |
| Typhimurium | Reptiles | Lizard - Bearded dragon, Other reptile - Snake, Turtle - pet at home, Turtle - Turtle | Child daycare/preschool, Other | 33 | 6 | 18.2 | 8.6 - 34.4 |
| Uganda | Birds | Other poultry - Turkey | Other | 1 | 1 | 100 | 20.7 - 100 |
| unknown | Mammals | Cattle - Calf | School/college/university | 3 | 1 | 33.3 | 6.1 - 79.2 |
| unknown | NA | NA | Other | 3 | 2 | 66.7 | 20.8 - 93.9 |

**Table S2.** C**haracteristics of the network consisting of serovars, exposure sources, and settings.**

| **Variable Name** | **Node** | **Degree** | **Betweenness** | **Closeness** | **Clustering Coefficient** | **Community** |
| --- | --- | --- | --- | --- | --- | --- |
| Enteritidis | SEN | 2 | 0 | 0.0094 | 1.00 | 1 |
| I 4,[5],12:i:- | STM | 2 | 0 | 0.0075 | 1.00 | 2 |
| Typhimurium | STY | 5 | 8 | 0.0129 | 0.40 | 2 |
| Birds | BRD | 3 | 3 | 0.0106 | 0.67 | 1 |
| Mammals | MAM | 4 | 2 | 0.0094 | 0.50 | 2 |
| NA | NAA | 0 | 0 | NA | 0.00 | 3 |
| Reptiles | RPT | 2 | 2 | 0.0113 | 1.00 | 2 |
| Agricultural feed store | AFS | 0 | 0 | NA | 0.00 | 4 |
| Child daycare/preschool | DAY | 0 | 0 | NA | 0.00 | 5 |
| Fairground | FRG | 0 | 0 | NA | 0.00 | 6 |
| Farm/dairy/agricultural settings | FAR | 2 | 5 | 0.0110 | 1.00 | 2 |
| Hospital | HOS | 0 | 0 | NA | 0.00 | 7 |
| Other | OTH | 6 | 4 | 0.0100 | 0.33 | 2 |
| School/college/University | SCH | 0 | 0 | NA | 0.00 | 8 |
| Veterinary clinic | VET | 0 | 0 | NA | 0.00 | 9 |

**Table S3. Overall Eigenvalues and explained variance of MCA dimensions for NTS serovars Typhimurium, I 4,[5],12:i:-, and Enteritidis**

| Dimensions | Eigenvalue | Variance Percent | Cumulative Variance Percent |
| --- | --- | --- | --- |
| Dim.1 | 0.252 | 25.23 | 25.235 |
| Dim.2 | 0.198 | 19.84 | 45.080 |
| Dim.3 | 0.162 | 16.16 | 61.246 |
| Dim.4 | 0.102 | 10.24 | 71.492 |
| Dim.5 | 0.099 | 9.916 | 81.408 |
| Dim.6 | 0.077 | 7.679 | 89.087 |
| Dim.7 | 0.071 | 7.054 | 96.142 |
| Dim.8 | 0.033 | 3.295 | 99.436 |
| Dim.9 | 0.006 | 0.564 | 100.00 |
| Dim.10 | 0.000 | 0.000 | 100.00 |

**Table S4. Contributions of Variables to the dimensions of the MCA for NTS serovars Typhimurium, I 4,[5],12:i:-, and Enteritidis**

| Variable Name | Dimension 1 | Dimension 2 | Dimension 3 | Dimension 4 | Dimension 5 |
| --- | --- | --- | --- | --- | --- |
| SEN_0 | 4.336 | 0.448 | 0.172 | 0.000 | 0.4930 |
| SEN_1 | 19.94 | 2.062 | 0.792 | 0.002 | 2.2660 |
| STM_0 | 1.360 | 8.933 | 0.000 | 0.004 | 0.6420 |
| STM_1 | 3.716 | 24.41 | 0.000 | 0.011 | 1.7540 |
| STY_0 | 1.773 | 22.42 | 0.320 | 0.011 | 3.9120 |
| STY_1 | 1.429 | 18.08 | 0.258 | 0.009 | 3.1550 |
| BRD_0 | 7.238 | 0.010 | 0.084 | 0.039 | 6.3610 |
| BRD_1 | 18.09 | 0.030 | 0.211 | 0.097 | 15.902 |
| MAM_0 | 10.84 | 1.850 | 8.894 | 0.000 | 0.0690 |
| MAM_1 | 9.398 | 1.61 | 7.708 | 0.000 | 0.060 |
| RPT_0 | 0 | 0.741 | 3.582 | 0.037 | 6.800 |
| RPT_1 | 0.002 | 4.444 | 21.49 | 0.225 | 40.80 |
| AFS_0 | 0.466 | 0.003 | 0.225 | 1.263 | 0.110 |
| AFS_1 | 12.576 | 0.092 | 6.084 | 34.08 | 2.978 |
| OTH_0 | 0.976 | 6.534 | 21.81 | 0.020 | 5.802 |
| OTH_1 | 0.325 | 2.178 | 7.271 | 0.007 | 1.934 |
| SCH_0 | 0.111 | 0.001 | 0.052 | 1.146 | 0.079 |
| SCH_1 | 6.132 | 0.044 | 2.853 | 63.04 | 4.349 |
| VET_0 | 0.046 | 0.217 | 0.65 | 0.000 | 0.090 |
| VET_1 | 1.233 | 5.863 | 17.54 | 0.001 | 2.442 |

**Table S5. Individual outbreak Contributions to the dimensions of the MCA for NTS serovars Typhimurium, I 4,[5],12:i:-, and Enteritidis**

| Outbreak ID | Dim 1 | Dim 2 | Dim 3 | Dim 4 | Dim 5 |
| --- | --- | --- | --- | --- | --- |
| 1 | 1.170 | 3.790 | 0.041 | 0.001 | 0.075 |
| 2 | 1.170 | 3.790 | 0.041 | 0.001 | 0.075 |
| 3 | 4.515 | 0.625 | 0.264 | 0.010 | 0.842 |
| 4 | 0.043 | 2.180 | 5.389 | 0.022 | 3.350 |
| 5 | 0.111 | 0.951 | 4.664 | 0.049 | 9.102 |
| 6 | 0.915 | 0.255 | 0.002 | 0.002 | 0.833 |
| 7 | 0.915 | 0.255 | 0.002 | 0.002 | 0.833 |
| 8 | 0.915 | 0.255 | 0.002 | 0.002 | 0.833 |
| 9 | 0.043 | 2.180 | 5.389 | 0.022 | 3.350 |
| 10 | 0.915 | 0.255 | 0.002 | 0.002 | 0.833 |
| 11 | 0.540 | 1.326 | 1.822 | 0.000 | 0.003 |
| 12 | 0.184 | 1.215 | 0.222 | 0.000 | 0.150 |
| 13 | 0.915 | 0.255 | 0.002 | 0.002 | 0.833 |
| 14 | 1.170 | 3.790 | 0.041 | 0.001 | 0.075 |
| 15 | 1.170 | 3.790 | 0.041 | 0.001 | 0.075 |
| 16 | 0.378 | 2.674 | 0.612 | 0.006 | 1.062 |
| 17 | 4.515 | 0.625 | 0.264 | 0.010 | 0.842 |
| 18 | 0.547 | 0.666 | 0.891 | 0.023 | 4.916 |
| 19 | 0.547 | 0.666 | 0.891 | 0.023 | 4.916 |
| 20 | 0.547 | 0.666 | 0.891 | 0.023 | 4.916 |
| 21 | 0.000 | 4.507 | 1.025 | 0.039 | 7.249 |
| 22 | 0.540 | 1.326 | 1.822 | 0.000 | 0.003 |
| 23 | 1.170 | 3.790 | 0.041 | 0.001 | 0.075 |
| 24 | 4.515 | 0.625 | 0.264 | 0.010 | 0.842 |
| 25 | 1.386 | 0.017 | 3.577 | 0.040 | 9.796 |
| 26 | 0.915 | 0.255 | 0.002 | 0.002 | 0.833 |
| 27 | 0.540 | 1.326 | 1.822 | 0.000 | 0.003 |
| 28 | 0.540 | 1.326 | 1.822 | 0.000 | 0.003 |
| 29 | 0.378 | 2.674 | 0.612 | 0.006 | 1.062 |
| 30 | 4.515 | 0.625 | 0.264 | 0.010 | 0.842 |
| 31 | 0.924 | 2.140 | 0.133 | 0.011 | 1.837 |
| 32 | 0.915 | 0.255 | 0.002 | 0.002 | 0.833 |
| 33 | 0.547 | 0.666 | 0.891 | 0.023 | 4.916 |
| 34 | 0.039 | 0.738 | 0.669 | 0.002 | 0.665 |
| 35 | 15.47 | 0.088 | 4.613 | 64.59 | 4.312 |
| 36 | 0.043 | 2.180 | 5.389 | 0.022 | 3.350 |
| 37 | 1.170 | 3.790 | 0.041 | 0.001 | 0.075 |
| 38 | 1.170 | 3.790 | 0.041 | 0.001 | 0.075 |
| 39 | 0.915 | 0.255 | 0.002 | 0.002 | 0.833 |
| 40 | 0.043 | 2.180 | 5.389 | 0.022 | 3.350 |
| 41 | 0.043 | 2.180 | 5.389 | 0.022 | 3.350 |
| 42 | 1.170 | 3.790 | 0.041 | 0.001 | 0.075 |
| 43 | 0.739 | 1.690 | 2.285 | 0.005 | 1.291 |
| 44 | 1.556 | 5.818 | 14.178 | 0.000 | 1.211 |
| 45 | 1.170 | 3.79 | 0.0410 | 0.001 | 0.075 |
| 46 | 0.547 | 0.666 | 0.8910 | 0.023 | 4.916 |
| 47 | 0.540 | 1.326 | 1.8220 | 0.000 | 0.003 |
| 48 | 1.170 | 3.79 | 0.0410 | 0.001 | 0.075 |
| 49 | 15.86 | 0.091 | 4.9180 | 17.46 | 1.477 |
| 50 | 1.170 | 3.79 | 0.0410 | 0.001 | 0.075 |
| 51 | 1.556 | 5.818 | 14.178 | 0.000 | 1.211 |
| 52 | 15.86 | 0.091 | 4.9180 | 17.46 | 1.477 |
| 53 | 0.001 | 2.267 | 0.2410 | 0.000 | 0.002 |
| 54 | 0.547 | 0.666 | 0.8910 | 0.023 | 4.916 |
| 55 | 0.184 | 1.215 | 0.2220 | 0.000 | 0.150 |
| 56 | 0.915 | 0.255 | 0.0020 | 0.002 | 0.830 |

**Supplementary Figures**


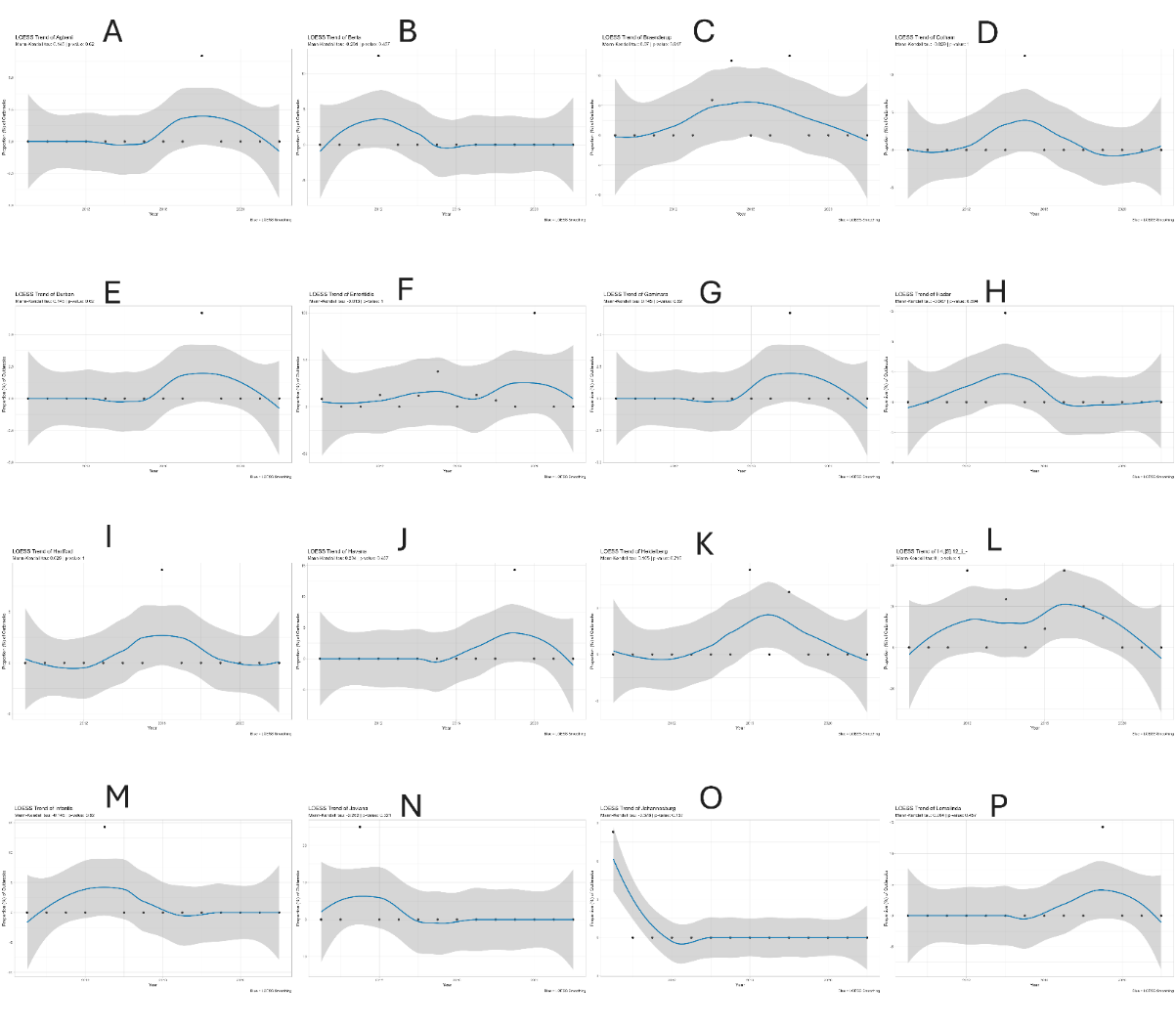


**Figure S1.** Trend analysis of *Salmonella enterica* serovar proportions in animal contact-related outbreaks, U.S., 2009–2022.

A-Agbeni, B-Berta, C-Braenderup, D-Cotham, E-Durban, F-Enteritidis, G-Gaminara, H-Hadar, I-Hartford, J-Havana, K-Heidelberg, L-I 4,[5],12:i:-, M-Infantis, N-Javiana, O-Johannesburg, and P-Lomalinda. The LOESS plot curve depicts changes in the serovar outbreak proportion over time, while the shaded area on the plot reflects the 95% confidence interval. The MK test tau values on the plot revealed the increasing/decreasing trend, and the p-value depicts the significance of the trend.


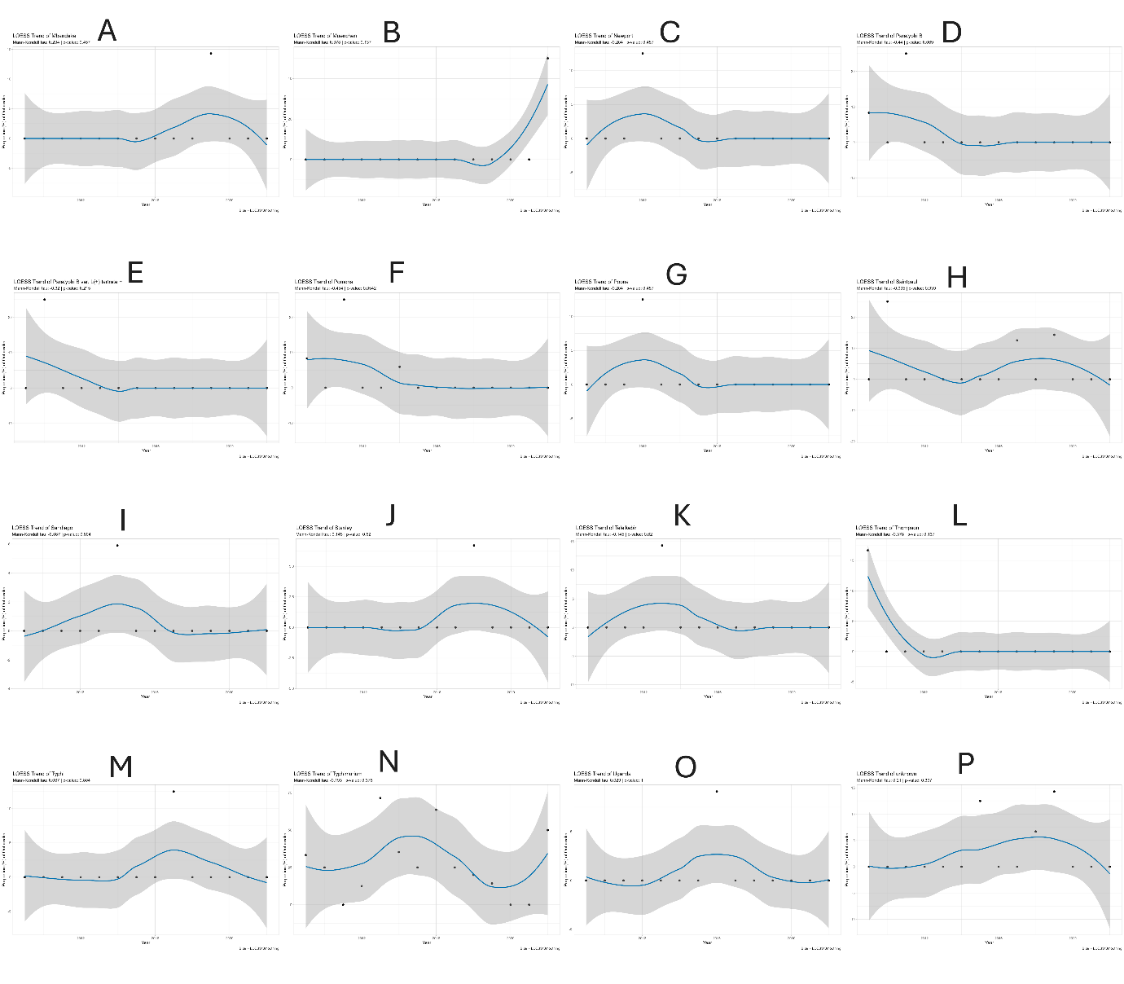


**Figure S2.** Trend analysis of Salmonella enterica serovar proportions in animal contact-related outbreaks, U.S., 2009–2022. A-Mbandaka, B-Muenchen, C-Newport, D-Paratyphi B, E-Paratyphi B var. L(+) tartrate +, F-Pomona, G-Poona, H-Saintpaul, I-Sandiego, J-Stanley, K-Telelkebir, L-Thompson, M-Typhi, N-Typhimurium, O-Uganda, and P-Unknown**.** The LOESS plot curve in each figure depicts changes in the serovar outbreak proportion over time, while the shaded area on the plot reflects the 95% confidence interval. The MK test tau values on the plot revealed the increasing/decreasing trend, and the p-value depicts the significance of the trend.
